# Reproductive Physiology of Halophytes: Current Standing

**DOI:** 10.3389/fpls.2018.01954

**Published:** 2019-01-09

**Authors:** Fang Yuan, Jianrong Guo, Sergey Shabala, Baoshan Wang

**Affiliations:** ^1^Shandong Provincial Key Laboratory of Plant Stress, College of Life Sciences, Shandong Normal University, Jinan, China; ^2^Department of Horticulture, Foshan University, Foshan, China; ^3^College of Sciences and Engineering, University of Tasmania, Hobart, TAS, Australia

**Keywords:** abscisic acid, dimorphism, osmolyte, reproductive biology, salt tolerance, salinity, survival

## Abstract

**Background:** Halophytes possess efficient salt-tolerance mechanisms and can complete their life cycles in naturally saline soils with NaCl contents exceeding 200 mM. While a significant progress have been made in recent decades elucidating underlying salt-tolerance mechanisms, these studies have been mostly confined to the vegetative growth stage. At the same time, the capacity to generate high-quality seeds and to survive early developmental stages under saline conditions, are both critically important for plants. Halophytes perform well in both regards, whereas non-halophytes cannot normally complete their life cycles under saline conditions.

**Scope:** Research into the effects of salinity on plant reproductive biology has gained momentum in recent years. However, it remains unclear whether the reproductive biology of halophytes differs from that of non-halophytes, and whether their reproductive processes benefit, like their vegetative growth, from the presence of salt in the rhizosphere. Here, we summarize current knowledge of the mechanisms underlying the superior reproductive biology of halophytes, focusing on critical aspects including control of flowering time, changes in plant hormonal status and their impact on anther and pollen development and viability, plant carbohydrate status and seed formation, mechanisms behind the early germination of halophyte seeds, and the role of seed polymorphism.

**Conclusion:** Salt has beneficial effects on halophyte reproductive growth that include late flowering, increased flower numbers and pollen vitality, and high seed yield. This improved performance is due to optimal nutrition during vegetative growth, alterations in plant hormonal status, and regulation of flowering genes. In addition, the seeds of halophytes harvested under saline conditions show higher salt tolerance than those obtained under non-saline condition, largely due to increased osmolyte accumulation, more optimal hormonal composition (e.g., high gibberellic acid and low abcisic acid content) and, in some species, seed dimorphism. In the near future, identifying key genes involved in halophyte reproductive physiology and using them to transform crops could be a promising approach to developing saline agriculture.

## Introduction

Coastal salt marshes and inland lakes contain significant amounts of salt, and inappropriate agricultural irrigation has also created large areas of saline environment (Galvan-Ampudia and Christa, 2011; Yuan et al., 2016a), contaminating both freshwater reservoirs and soil, particularly in arid and semiarid climatic zones (Rozema and Flowers, 2008). While soil salinization threatens the life cycle of most plants, approximately 1% of known terrestrial plant species flourish in saline conditions and are referred to as halophytes (Rozema and Flowers, 2008). The definition of a halophyte is somewhat subjective and varies in the literature. The most salt-tolerant halophytes can complete their life cycles in soils containing concentrations of NaCl, equal to, or even exceeding, that of seawater, e.g., 500 mM (Shabala, 2013; Song and Wang, 2015). However, the conventional definition used in the literature is that plants possess halophytism if they are able to survive and reproduce at NaCl concentrations exceeding 200 mM NaCl (Flowers and Colmer, 2008; Flowers et al., 2015; Santos et al., 2015; Yuan et al., 2016a). Based on mechanisms employed to deal with salinity load, halophytes can be divided into three categories: euhalophytes [which can actively compartmentalize toxic ions into their vacuoles, such as *Suaeda salsa* (Song et al., 2011; Li X. et al., 2012)], recretohalophytes [which directly secret salt outside by salt-secretory structures, e.g., *Chenopodium quinoa* (Shabala et al., 2014) and *Limonium bicolor* (Yuan et al., 2016a, 2018)], and pseudo-halophytes [which can exclude rather than absorb salt in their roots, such as *Avicennia officinalis* (Krishnamurthy et al., 2014)].

**FIGURE 1:**
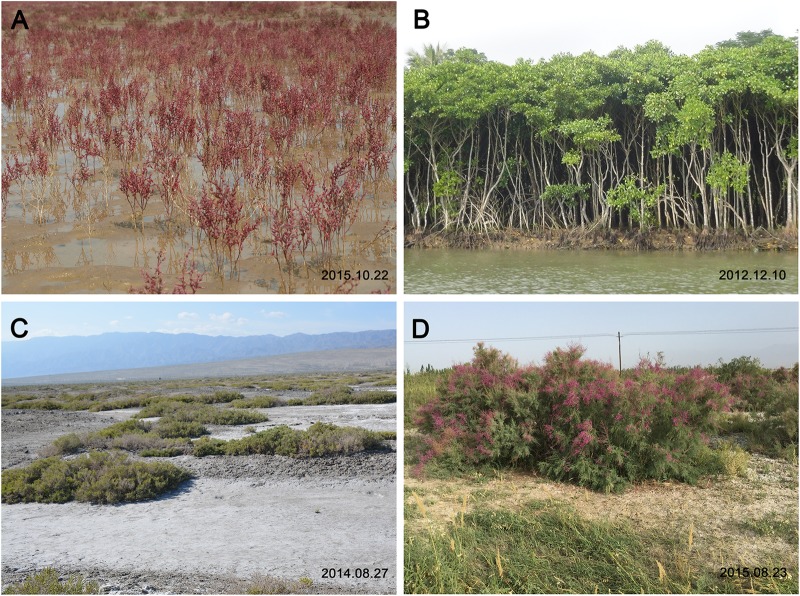
Typical saline habitats and halophytes across the globe. **(A)** *Suaeda salsa* grows well and forms a community in the intertidal zone, which is flooded regularly by seawater. The photograph was taken in the Yellow River Delta (N 37°25′; E 118°54′). **(B)** Mangroves grow in a sea water. The photograph was taken in the Dongzhai Harbor National Nature Reserve in Haikou (N 19°51′–20°1′; E 110°32′–110°37′). **(C)** Halophyte communities (*Nitraria tangutorum* and *Alhagi sparsifolia*) live in inland saline soil. The photograph was taken near Ebinur Lake (N 44°50′; E 82°45′) in North Xinjiang. **(D)** Photograph of *Tamarix gallica* in Luntai County (N 41°47′; E 84°14′) in South Xinjiang.

Heredity determines the geographical distribution of halophytes and non-halophytes and their responses to salinity (Ding et al., 2010a,b; Zhao K.F. et al., 2010; Guo et al., 2012a,b). The two groups show distinct differences in their maximum salt tolerance, and tend to form natural halophytic and non-halophytic populations in saline soil and non-saline alkali soil, respectively (Chen et al., 2010; Sui et al., 2010; Zhao S.Z. et al., 2010; Sun et al., 2013). Across the globe, halophytes are found in two typical kinds of saline environments: intertidal zones (Figures 1A,B) and inland saline soils (Figures 1C,D). They can grow to maturity and complete their life cycles in seawater or in highly saline soil (Chen et al., 2016); examples include mangrove (Tan et al., 2013), *S. salsa* (Song et al., 2008), and *L. bicolor* (Feng et al., 2014, 2015; Yuan et al., 2016b). In contrast, non-halophytes (such as most crop plants) are found only in non-saline soils. Though non-halophytes have also evolved various strategies to respond to salt stress, their growth declines sharply with increased NaCl concentration, whereas halophytes can benefit from higher salt concentrations, within reason, and show an optimal growth in the presence of significant amounts of NaCl: e.g., 200 mM for *S. salsa* (Yang et al., 2010) and *Suaeda fruticosa* (Khan et al., 2000), 150 mM for *Chenopodium quinoa* (Shabala et al., 2012), and 100 mM for *Cakile maritima* (Debez et al., 2004).

Numerous papers have been published dealing with various aspects of halophyte physiology (Shabala, 2013; Shabala et al., 2014; Song and Wang, 2015; Yuan et al., 2016b; Dassanayake and Larkin, 2017; Leng et al., 2018; Liu Q. et al., 2018), biochemistry and molecular biology (Kirch et al., 2000; Oh et al., 2015; Himabindu et al., 2016; Ozfidankonakci et al., 2016), ecology (Flowers and Colmer, 2008; Rozema and Flowers, 2008), and evolution (Flowers et al., 2010). Moreover, the practical use of halophytes in saline agriculture has been actively advocated (Ruan et al., 2010). All these papers have provided insightful suggestions about the mechanisms underlying the superior vegetative growth of halophytes under saline conditions.

While vegetative growth is important throughout the life of a plant, the capacity to set seeds in a hostile environment, such as saline soil, is equally critical for completing the life cycle. During the reproductive phase of plant growth, the meristem growth is almost always determinate, although the extent of determinacy depends on the inflorescence architecture (Kwiatkowska, 2008). Salinity stress strongly affects reproductive growth. Non-halophytes cannot naturally form seeds under saline conditions, and therefore fail to complete their life cycles. The reproductive growth is an essential stage of the plant life cycle, and early germination is equally important to establishing a population in a saline soil. While research into the effects of salinity on plant reproductive biology has been gaining momentum in recent years, it remains unclear whether the reproductive biology of halophytes differ from that of non-halophytes, and whether aspects of halophyte biology relevant to reproduction traits benefit from the presence of salt, like those related to vegetative growth. In the current review, we focus on the reproductive biology of halophytes and non-halophytes grown in saline environment.

## Halophyte Reproductive Growth Under Saline Conditions

Being grown under high-salinity conditions, halophytes often show increased flower number, decreased sterility, and high seed quality (Guo et al., 2018). At the same time, salinity markedly reduces the flower and seed numbers of non-halophytes grown under same conditions (Sohrabi et al., 2008; Khan et al., 2015). This difference may be attributed to the limited resource allocation to flowers and developing seeds in non-halophytes, which results in a lower fertilization efficiency and less seed formation (Ledesma and Sugiyama, 2005; Guo et al., 2015).

### Halophyte Flowering Time Benefits From Salinity

Halophytes typically show delayed flowering time under appropriate salinity. For example, treating plants of the euhalophyte *S. salsa* with 400 mM NaCl significantly delays the time of the first flowering as compared to that in *S. salsa* not subjected to saline treatment (Guo et al., 2018). In contrast, in non-halophytes, salt stress can often cause an early flowering (Lee et al., 1994) and abortion of flower buds (Sulpice et al., 2003). Halophytes can also undergo longer flowering periods (florescence) under saline as compared to non-saline conditions (Guo et al., 2018).

The underlying mechanisms controlling flowering time may be explained by genes, domestication, and sustainable productivity (Cockram et al., 2007), but whether these factors are affected by salt has been largely not investigated in halophytes. Some clues may be found in studies from *Arabidopsis thaliana* (Arabidopsis). It was shown that in this species, a good flower onset may benefit from the synthesis of the osmoregulator glycine betaine (Sulpice et al., 2003). Recent studies have investigated several genes related to the flowering time and their protein products. BFT (BROTHER OF FT AND TFL1), a floral repressor, participates in the inhibition of flowering under high salinity by competing with FT (FLOWERING LOCUS T) for binding to the FD transcription factor (Ryu et al., 2014). CDKG2 (CYCLIN-DEPENDENT KINASE G2) also plays a role in the control of flowering time under saline conditions (Ma et al., 2015). In addition, DDF1 (DWARF AND DELAYED-FLOWERING 1) is also involved in controlling late flowering, and its expression has been found to always accompany gibberellic acid (GA) synthesis (Magome et al., 2008).

### Halophyte Anther and Pollen Maintain High Vitality in Saline Conditions Due to Altered Hormonal Status

Male reproductive development is extremely sensitive to salt stress as a result of a variety of factors associated with cytoskeletal alterations, tapetal irregularities, altered sugar utilization, and meiotic defects or abortion (Nico and Danny, 2014). Pollen development involves a series of stages, including specification of stamen identity, archesporial cell initiation, anther cell establishment, and meiosis (Zhao D. et al., 2003). In most species, the more mature pollen is, the more susceptible to abiotic stress it is. The anther and pollen vitality is an indispensable factor inducing sterility of non-halophytes under salinity stress. However, in *S. salsa*, the pollen number and pollen activity under saline conditions are higher than, or equal to those, under non-saline conditions, implying that high NaCl concentration markedly improves the reproductive capacity (Guo et al., 2018).

The mechanisms behind the decreased pollen vitality in non-halophytes under salinity stress remain to be identified. In rice and Arabidopsis, the plant hormone gibberellin (GA) participates in stamen development and tapetum function (Plackett et al., 2011), and is tightly intertwined with the secretion of callose and the synthesis and secretion of proteins and lipids for the pollen coat into the anther locule (Parish and Li, 2010). In addition to GA’s direct roles in the pollen mother cells, a loss of GA signal in the tapetum could indirectly block further development of the gametophytes. In a late stamen development, GA signal transduction acts partially through a jasmonic acid (JA) signal via regulation of JA biosynthesis (Plackett et al., 2011). However, the question of whether GA maintains a stable level in halophytes requires further investigation.

Salinity can also promote senescence and induce the production of two other stress-related hormones, ethylene and abcisic acid (ABA) (Nandwal et al., 2007), which can increase the number of aborted flowers. Reproductive growth is coordinated with vegetative growth based on the balance or homeostasis between ethylene and its receptors. When non-halophytes suffer from salinity stress, enhanced ethylene production leads to small rosettes and relatively early flowering, limiting energy and resource utilization for production of seeds (Cao et al., 2008). In halophytes, NaCl participates in the conversion of the precursor l-aminocyclopropane-l-carboxylic acid (ACC) to ethylene (Chrominski et al., 1986, 1988), and this process is enhanced under salinity stress in *Allenrolfea occidentalis* (Chrominski et al., 1989).

### Halophyte Seed Formation and Yield Benefit From the Presence of Salt

**FIGURE 2:**
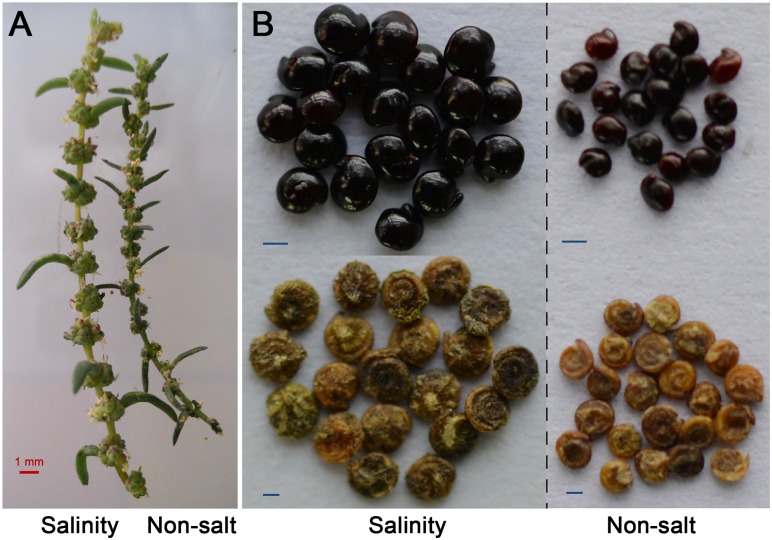
Flower number **(A)** and seed weights **(B)** of *Suaeda salsa* grown with NaCl were markedly enhanced compared with those of *S. salsa* grown without NaCl. Data shown in panel **A** are from our laboratory, bar = 1 mm. Seed photos in **(B)** originated (Guo et al., 2018), bar = 250 μm. Treatments were applied to the sand in which the seeds were sown and then continued until seed maturity. The seeds were watered twice a day with either a no-NaCl solution (Hoagland nutrient solution, as control) or 200 mM of NaCl dissolved in Hoagland nutrient solution.

A high positive correlation between leaf area and yield in the presence of salt exists in many crop species (Richards, 1992). NaCl treatment did not specifically decrease the development of reproductive organs in these species, and the production losses caused by high salinity may result from a reduction in flower production and/or a decrease in the flower fertility (Khatun and Flowers, 1995). Interestingly, our recent study found that the halophyte *S. salsa* produces a greater weight of seeds under high salinity than without saline treatment (Figure 2B; Guo et al., 2018); the same phenomenon has been observed in *Suaeda corniculata* (Yang et al., 2017). Similarly, in the halophyte *C. maritima*, seed production is stimulated by 50 to 100 mM NaCl as compared to a no-salt control treatment (Debez et al., 2004). Another halophyte, *Salicornia bigelovii*, displayed high seed yield and biomass production when irrigated with seawater (Glenn et al., 1998). Its increased seed yield was mainly due to increased flower number (Figure 2A) and reduced abortion ratio, which may be related to an increase in pollen vitality or stigma receptivity (Guo et al., 2015).

### Higher Carbohydrate Supply Improves Halophyte Reproductive Growth

In rice, agronomic characteristics related to grain yield show significant decreases at 50 mM NaCl, mainly due to limitation in the soluble carbohydrate translocation in spikelets and a significant inhibition of starch synthase activity (Abdullah et al., 2010). The situation is rather different in halophytes. The higher net photosynthetic rates and adequate carbon supply under saline conditions promote the reproductive growth of *S. salsa* and improve the flower fertility and seed size, compared to non-saline conditions (Guo et al., 2018). Thus, the superior reproductive growth of halophytes may be fundamentally attributable to their better resource allocation to flower and developing seeds, as compared with non-halophytes (Monteiro et al., 2003; Wang et al., 2017). In non-halophytes, maternal plants grown under saline conditions are unable to provide sufficient nutrients to the floral organ, leading to poor reproductive growth or reproductive failure.

### Possible Genes Involved in Salt Response During Reproductive Growth

Plant reproductive development is controlled by multiple key regulators of floral identity. *APETALA1* (*AP1*)/*CAULIFLOWER* (*CAL*) and *LEAFY* (*LFY*) are redundantly activated by FT [reviewed by Ruiz-García et al. (1997); Komeda (2004); Blázquez et al. (2006)]. *FLC* (*FLOWERING LOCUS C*, a negative regulatory gene) and *FT* (a positive regulatory gene) are two important flowering-related and determined genes that regulate the induction of flowering (Lee, 2011; Xu et al., 2012). Signaling by EXCESS MICROSPOROCYTES1/TAPETUM DETERMINANT1 (EMS1/TPD1) determines cell fate during plant sexual reproduction (Jia et al., 2008). Kim and Park (2007) have found that the transcription factor NTL8 (NTM1-Like’s 8) regulates flowering time under salt stress by downregulating *FT*. EARLY FLOWERING3 (ELF3) may also be involved in salt tolerance (Sakuraba et al., 2017). CYCLIN-DEPENDENT KINASE G2 (CDKG2) negatively regulates flowering time in response to salinity stress (Ma et al., 2015). However, it should be kept in mind that all this knowledge comes from studies on non-halophytes and thus, cannot be directly extrapolated to halophytes without additional studies.

## Early Germination of Halophytes Under Saline Conditions

Seed formation is the end of a plant life cycle for annual plants, but also a new start for the next generation. From the perspective of generational reproduction and population formation, whether a new plant can survive, or a population can develop under saline conditions, is also determined by the germination process. In the next section, we therefore review current knowledge about the early survival of halophytes under saline conditions. Halophytes have evolved several strategies to cope with salinity during germination, including seed dormancy and heteromorphism, which are well described in previous reviews (Gul et al., 2013). Here we focus on the in-depth mechanisms that may explain the higher germination frequencies of halophytes as compared to non-halophytes under saline conditions.

### Halophytes Show Higher Seed Germination Than Non-halophytes at High Salinity

Halophytes can germinate at salinities that kill 99% of non-halophytes (Manousaki and Kalogerakis, 2011), indicating that they are more salt tolerant at the germination stage (Ungar, 1978). Though the seeds of both halophytes and non-halophytes are able to imbibe water from a saline substrate in a similar manner, their behavior is otherwise strikingly different (Malcolm et al., 2003). To date, the seeds of at least ten species of halophytes have been shown to have higher germination percentages at slightly elevated salinity (0.5%, around 50–90 mM) than in distilled water (Qu et al., 2007; Zhang et al., 2012). In the halophyte *C. maritima*, NaCl inhibits germination only at concentrations higher than 200 mM, mainly through an osmotic effect (which is fully reversible if the seeds are transferred to water) (Debez et al., 2004). This is consistent with the reported beneficial effects of salt on halophyte vegetative growth (Flowers and Colmer, 2008).

**FIGURE 3:**
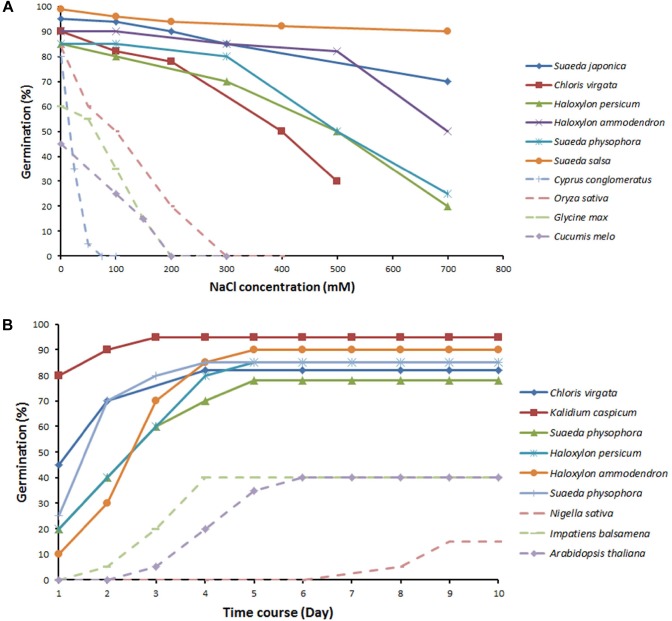
Effect of increasing salinity on the germination percentage **(A)** and the time course of germination percentage under 100–200 mM NaCl **(B)** in halophytes and non-halophytes. Halophytes (solid lines): *Suaeda japonica* (Yokoishi and Tanimoto, 1994), *Chloris virgata* (Zhang et al., 2012), *Kalidium capsicum* (Tobe et al., 2000), *Suaeda physophora* (Song et al., 2005), *Haloxylon persicum*, *Haloxylon ammodendron*, *Suaeda physophora* (Song et al., 2005), and *Suaeda salsa* (Song et al., 2008). Non-halophytes (dotted lines): *Cyperus conglomeratus* (Keblawy et al., 2011), *Oryza sativa* (Jiang et al., 2013), *Glycine max* (Neves et al., 2005), *Nigella sativa* (Papastylianou et al., 2017), *Cucumis melo* (Sohrabikertabad et al., 2013), *Impatiens balsamina* (Jiang et al., 2014), and *Arabidopsis thaliana* (Wilson et al., 2014).

The above notion is further illustrated by Figure 3, which shows the germination percentages of typical halophytes and non-halophytes under an NaCl concentration gradient and over time. Though beneficial effects of salt on germination have been reported for some halophytes (Debez et al., 2004), the majority of halophytes still show a decreased germination even under low concentration of salinity. Interestingly, the same salinity levels that can promoting vegetative growth [e.g., 200 mM in *S. salsa* in their natural habitats (Song et al., 2008) and 100 mM in *Plantago crassifolia* (Vicente et al., 2004)] suppress the seed germination of the same species. *Spergularia marina* even fails to germinate in the 2% NaCl treatment (Keiffer and Ungar, 1997). Thus, seed germination trait appears to be more sensitive to salt stress compared to vegetative growth. Nevertheless, halophytes still perform much better than non-halophytes at the germination stage. The germination percentages for non-halophytes decrease sharply at even low concentrations of NaCl, and some species fail to germinate above 100 mM NaCl. In contrast, halophytes maintain relatively high germination percentages under saline conditions, which decline slowly with increasing concentrations of NaCl. There is also a large genetic variability in germination ability/rate amongst halophytes grown under saline conditions. *S. salsa* maintains more than 90% of its germination percentage being exposed to 700 mM NaCl as compared to non-saline conditions (Figure 3A). *S. salsa* and *Kalidium capsicum* can rapidly reach more than 50% germination percentages in the first day, while other halophytes (*Suaeda physophora* and *Haloxylon persicum*) keep slowly increasing germination with time (Figure 3B). Comparative analysis of the time course of germination in 100–200 mM NaCl between halophytes and non-halophytes reveals that maximum germination of halophytes is achieved in the first 3 days.

Another advantage of halophytes compared with non-halophytes is that the former can easily get recovery when transferred to water or solutions of lower salinity. Similar to germination, the recovery phenomenon also varies between different halophytes. Some species show high recovery rate of full germination (reach the control), such as *Suaeda fruticosa* (Khan and Ungar, 1997), *P. crassifolia* (Vicente et al., 2004) and *Haloxylon recurvum* (Khan and Ungar, 1996). Other halophytes exhibit low or little recovery, e.g., *Zygophyllum simplex* (Khan and Ungar, 1997).

Inhibition of halophyte germination under high salt concentration can be alleviated by mild saline pretreatment. For example, seeds of the halophyte *Crithmum maritimum* that are pretreated with 50 mM NaCl show a higher germination percentage and faster germination at 500 mM NaCl than untreated seeds (Meot-Duros and Magné, 2008), and the same phenomenon has been observed for *Arthrocnemum macrostachyum* and *Sarcocornia fruticosa*, in which osmotic pretreatments (with salts such as NaCl and MgCl_2_) promote germination, doubling the germination percentage under saline conditions compared to that of plants pretreated with distilled water (Pujol et al., 2000). The germination of halophyte *P. crassifolia* can reach the control after salt priming (Vicente et al., 2004). Further details and mechanisms behind this osmopriming effect in halophytes have been reviewed by Gul et al. (2013).

### Halophytes Accumulate High Levels of Osmolytes During Seed Formation

Seeds of *S. salsa* harvested under saline conditions for three successive generations showed higher germination percentage than those harvested under non-saline conditions (Li W. et al., 2011; Guo et al., 2015), mainly as a result of the increased accumulation of osmoregulating substances (such as Na^+^, soluble sugars, and seed starch) in saline-grown seeds. These substances can reduce the water potential of seeds, thus contributing to quick imbibition under saline conditions.

The seed coat plays a significant role in maintaining seed viability under hypersaline conditions (Song et al., 2017). Lipid mobilization (as evidenced by a high transition ratio of phosphatidylglycerol to sulfoquinovosyldiacylglycerol) (Li X. et al., 2011), chlorophyll accumulation (Li X. et al., 2012; Zhou J.C. et al., 2016), soluble sugar accumulation, and sufficient storage of energy from photosynthesis in seeds (Rolletschek et al., 2003; Weber et al., 2005) all facilitate rapid germination under saline conditions (Song and Wang, 2015). Interestingly, in some halophytes, such as *Haloxylon ammodendron* and *Suaeda physophora*, chlorophyll is found in both dry and imbibed seeds (Zhang et al., 2010), regardless of whether they were treated with NaCl. In non-halophyte species, chlorophyll is found in some immature (Tasaki, 2008) but not in mature seeds. Increased chlorophyll concentration and oxygen production observed in the embryos of maturing *S. salsa* seeds may enhance the salt tolerance of the seeds and seedlings by changing the lipid composition of membranes (Zhou J.C. et al., 2016). It has also been suggested that nitrates provided to seeds by maternal plants may act as signaling molecules to enhance germination, enabling plant adaptation to saline environments (Song et al., 2016). It remains to be determined whether this observation can be extrapolated to all halophytes.

### Phytohormones Participate in Halophyte Early Germination

Plant hormones play a pivotal role in seed germination and seed formation under saline conditions (Wang et al., 2015). Studies of non-halophytes have indicated that the reduced seed germination of Arabidopsis under saline conditions is caused by alterations in plant hormonal status (Jung and Park, 2011). Several genes are strongly induced by NaCl and appear to be involved in the regulation of seed germination through ABA–GA crosstalk during salt stress (Yuan et al., 2011). In particular, the ABA level increases several fold under saline conditions due to the dramatic increase in expression of the genes *ABA-INSENSITIVE 3* (*ABI3*) and *ABA-INSENSITIVE 5* (*ABI5*), which in turn activates the ABA signaling pathway, resulting in inhibition of seed germination (Piskurewicz et al., 2008). Meanwhile, *REPRESSOR OF GA-LIKE2* (*RLG2*) transcription is also activated by salt or by the ABI3/ABI5 pathway, leading to inactivation of the GA signaling pathway, which further inhibits germination by blocking or limiting GA signaling (Yuan et al., 2011). In addition, NaCl has been observed to induce a negative regulation of GA and a positive biogenesis of ABA, which delays soybean seed germination (Shu et al., 2017). Mechanisms underlying osmopriming are also directly related to the ABA-GA network (Nakaune et al., 2012).

In addition, ethylene has been reported to promote seed germination of non-halophytes such as Arabidopsis and lettuce under salinity stress by upregulating the gene *ETHYLENE-INSENSITIVE 3* (*EIN3*) (Verma et al., 1973; Lin et al., 2013). It has been verified in 22 species of halophytes that the application of exogenous ethylene significantly promotes seed germination (Khan et al., 2009), especially during later stages of seed germination (such as radicle breaching of the seed coat) (Li and Tran, 2017). Thus, it appears that the genes related to ABA, GA, and ethylene may coordinate and participate in the response of halophytes to salt that allows them to maintain high germination rates. In non-halophytes, salt induces an increase in ABA and a decrease in GA in the embryo, thus preventing germination. It may be envisaged that in halophyte seeds, comparatively less ABA is released, more GA accumulates, and more ethylene synthesis occurs during germination, explaining their stronger performance under saline conditions. However, as only few studies have been performed in halophytes to explain the molecular mechanisms underlying their good germination under saline conditions, this hypothesis requires further confirmation.

### Ca^2+^ Is Involved in the Alleviation of Salt Toxicity

Salinity stress symptoms in plants can be ameliorated by exogenous Ca^2+^ application (Rengel, 1992b). Ca^2+^ participated in salt alleviation to some degree NaCl stress/toxicity for certain types of soils (Rengel, 1992a; Zhu, 2016), involved in enhancing seed germination of halophyte *Kalidium caspicum* (Tobe et al., 2002, 2004), facilitating radicle survival (Tobe et al., 2000) of *K. caspicum* and promoting polar vegetative growth (Mori and Schroeder, 2004) in non-halophyte species. The beneficial effects of Ca^2+^ treatment are mainly attributed to both reducing the rate of Na^+^ uptake by roots resulting from blockage of non-selective cation channels (NSCC) by millimolar Ca^2+^ concentrations (Demidchik and Tester, 2002), as well as to its ability to prevent NaCl-induced K^+^ leak via outward-rectifying channels (Shabala et al., 2007). Calcium also operates as a second messenger in both SOS (Mario and Zhu, 2009) and ABA signal pathway (Macrobbie, 2000; Sokolovski et al., 2008). Salt-tolerant genotypes appear to have a larger population of Ca^2+^-sensitive NSCC channels, compared with salt-sensitive counterparts such as barley (Chen et al., 2007). It remains to be answered if increased sensitivity to Ca^2+^ and/or its cross-talk with other signaling hormones may explain better performance of halophyte seeds under saline conditions.

### Physiology and Metabolic Profiles of Halophyte Dimorphic Seeds

Another possible explanation for the better seed germination and population establishment of halophytes under saline conditions is the presence of seed dimorphism (Liu R. et al., 2018), a feature of some annual halophyte species that can help plants adapt to a changing environment (Song and Wang, 2015). For example, soft brown seeds (with a higher germination percentage) and hard black seeds (dormant) are found in *S. salsa* (Li W. et al., 2008; Song et al., 2008) and *Suaeda acuminate* (Wang et al., 2012). Green seeds (long-winged type) and yellow seeds (short-winged type) are found in *Salsola komarovii* (Takeno and Yamaguchi, 1991), and the former show higher germination than latter. The same seed dimorphism is observed in *Salicornia europaea* with large seeds (formed from large flowers) and small seeds (small flowers) (Ungar, 1979; Gasparri et al., 2016). In general, the larger seeds are the main type showing rapid germination in most halophytes with dimorphic seeds, such as *Atriplex prostrata* (Wertis and Ungar, 1986), *Suaeda aralocaspica* (Wang et al., 2008), *S. salsa* (Song and Wang, 2015), and *S. europaea* (Orlovsky et al., 2016).

In addition to differences in germination percentages and appearance, dimorphic seeds also show differences in physiology and metabolic profiles related to the underlying mechanisms of their different behaviors. For example, the seed coats of brown *S. salsa* seeds contain more phenolics than black seeds (Xu et al., 2016), while the latter contain abundant waxes that can form a protective layer to shield the embryo from ion toxicity under saline conditions (Song et al., 2016). The black seeds of *S. corniculata* have an annual dormancy/non-dormancy cycle, while the brown seeds remain non-dormant. Salinity stress induces dormancy in black seeds, but decreases the viability of brown seeds (Cao et al., 2012). Thus, black seeds can better maintain their viability under long-time salinity than brown seeds (Song et al., 2017). In ecological terms, the black seeds can serve as a seed bank for long-term preservation in saline environments. Another difference between the two types of dimorphic seeds lies in their levels of endogenous hormones, including indole-3-acetic acid (IAA), free zeatin riboside (ZR), and ABA, which in *S. salsa* are much greater in brown seeds than in black seeds (Wang et al., 2015). These characteristics may help the species to ensure seedling establishment and population succession in variable saline environments. Finally, though the seeds of *S. corniculata* are collected from two distant population (F_0_), the descendants (F_1_ and F_2_) still kept phenotypic differences regardless of whether grown in low or high salinity, indicating that the traits of dimorphic seeds are genetically determined and that soil salinity only plays an ecological role in influencing heteromorphic seed production (high salinity results in fewer seeds and more non-dormant brown seeds) (Yang et al., 2017). Recently, Xu et al. (2017) used dimorphic seeds of *S. salsa* to perform differential expression analysis by transcriptome and identified a series of genes related to embryo development, fatty acid metabolism, osmoregulatory substances, and plant hormones that may regulate seed dormancy or germination. In this context, experiments with dimorphic seeds are a highly promising tool to reveal the mechanism of seed germination competence in halophytes.

## Perspectives

The reproductive growth and early germination are two critical processes that all plants must carry out, in order to survive under saline conditions, since only plants that can reproduce and germinate have a chance to complete their life cycle and establish descendant populations. Halophytes perform well in both respects under saline conditions. The current review mainly pay attention to the majority of halophytes that show promoted reproductive growth; however, it is worth noting that not all halophytes show the stimulation of growth or germination at low or moderate salinities, and some especial exceptions exist in different germplasms of the same species. For example, one accession (from Tabarka, Tunisian) of halophyte *Cakile maritime* behaves limited growth under 100 mM NaCl (Ben Amor et al., 2010), while other accessions of this species have enhanced biomass (Ksouri et al., 2007).

In the future, analyzing the in-depth mechanisms of salt tolerance in halophytes, isolating the unique genes involved, and creating new salt-tolerant plants by genetic engineering represents a promising approach toward developing saline agriculture. However, few reports have uncovered the key genes involved in the reproductive growth and early germination of halophytes. To date, certain salt-tolerance genes involved in vegetative growth have been cloned, and many key genes have been transformed into non-halophytes to verify their functions (Table 1). The maximum salt concentration tolerated by such transgenic plants was 400 mM (Li W. et al., 2011; Jha et al., 2012). Heterologous expression of halophytic salt-tolerance genes indeed improves the salt resistance of non-halophytes to a certain degree; however, no reports have demonstrated the stable expression of such genes in the next generation. The reason for this lies in a fact that the targeted traits were related to vegetative growth but not to seed formation or early germination. As a result, transgenic plants grew reasonably well in controlled condition but could not complete their life cycle in saline land.

**Table 1:** Cloned halophyte genes related to salt tolerance and their effects after transformation into non-halophytes.

Halophyte species	Gene	Probable function of gene product in salt tolerance	Effect when transformed into non-halophyte	Reference
*Aeluropus littoralis*	*AlNHX*	Vacuolar-type Na^+^/H^+^ antiporter	Transgenic tobacco overexpression lines had high salt tolerance (400 mM NaCl) and compartmentalized more Na^+^ in the roots to maintain a relatively high K^+^/Na^+^ ratio in the leaves.	Zhang et al., 2008
*Kalidium foliatum*	*KfVP1*	H^+^-pyrophosphatase	Transgenic Arabidopsis showed more vigorous growth than the wild type and accumulated more Na^+^ in the leaves in 120 mM NaCl	Yao et al., 2012
*Salicornia brachiata*	*SbASR-1*	Abscisic acid stress ripening-1	Transgenic T_0_ tobacco seeds showed better germination and seedling growth than the wild type in 400 mM NaCl	Jha et al., 2012
	*SbSOS1*	Plasma membrane Na^+^/H^+^ antiporter	Transgenic tobacco showed high seed germination and a high degree of salt tolerance in 200 mM NaCl	Yadav et al., 2012
	*SbpAPX*	Peroxisomal ascorbate peroxidase	Transgenic tobacco showed enhanced salt and drought tolerance, with enhanced vegetative growth and higher germination rates than the wild type in 300 mM NaCl	Singh et al., 2014
*Salsola soda*	*SsNHX1*	Putative vacuolar Na^+^/H^+^ antiporter	Transgenic *Medicago sativa* grew in high concentrations of NaCl (up to 400 mM) as a result of improved Na^+^ sequestration in vacuoles	Li W. et al., 2011
*Sesuvium portulacastrum*	*SpAQP1*	Aquaporin-related protein whose expression is induced by salt	Transgenic tobacco had increased activity of antioxidative enzymes, and enhanced seed germination and root growth in 200 mM NaCl	Chang et al., 2016
*Spartina alterniflora*	*SaVHAc1*	A vacuolar ATPase subunit c1	Transgenic rice showed early stomata closure and increased K^+^/Na^+^ ratio	Niranjan et al., 2012
*Suaeda salsa*	*SsVP*	Vacuolar H^+^-pyrophosphatase	Higher salt tolerance in transgenic Arabidopsis was related to higher activities of V-ATPase and V-PPase	Guo et al., 2006
	*SsHKT1;1*	High-affinity K^+^ transporter	Transgenic Arabidopsis showed enhanced salt tolerance and increased shoot K^+^ concentration	Shao et al., 2014
	*Ss.sAPX*	Stroma ascorbate peroxidase	Transgenic Arabidopsis overexpression lines had increased germination rates, cotyledon growth, and survival under saline conditions	Li K. et al., 2012
*Suaeda asparagoides*	*SaDhn* and *SaRBP1*	Dehydrin and RNA-binding protein	Transgenic yeast overexpression lines showed enhanced tolerance to osmotic, freezing, and heat-shock stresses	Ayarpadikannan et al., 2012
*Zoysia matrella*	*ZmVP1*	A type I VP homolog gene induced by salt	Transgenic Arabidopsis grew more vigorously than the wild type in 300 mM NaCl because of higher activities of V-ATPase and V-PPase	Chen et al., 2015


Therefore, to improve salinity tolerance in non-halophytes, key genes related to seed germination and formation should be also targeted and pyramided in the transformed plants. The utility of this approach was confirmed in a preliminary fashion by an assessment of Arabidopsis transformed with the gene *coda* involved in reproductive growth, which showed improved reproductive growth due to the resulting accumulation of the osmoregulator glycine betaine, especially in flowers and siliques (Sulpice et al., 2003).

Until now, with a single exception of *KcNHX* in *Karelinia caspica* (Liu L. et al., 2012), all salt-tolerance genes cloned in halophytes have been heterologously expressed in non-halophytes to illustrate their function. The usefulness of this approach is jeopardized by the fact that salinity tolerance is a complex trait that cannot be controlled by a single or a few genes, prompting a need for a pyramiding approach and targeting gene networks. This results in a need to obtain genetic evidence for the functional role of specific gene(s) in the “native” (i.e., halophyte) systems. The recent development of transformation systems for certain halophytes (Ishimaru, 1999; Sun and Hong, 2012; Yuan et al., 2014) opens prospects for validating the functions of the key halophyte genes by knockout, CRISPR-cas9, or overexpression studies in halophytes themselves. Moreover, many halophytes such as *S. europaea* is a promising crop (crop adapted halophyte), and present on food market as a tea, juice, powder, etc. (Gunning, 2016). Thus, the domestication of existing halophytic plants is another approach that should be considered for developing crops that can grow under saline conditions.

Given the paucity of the existing data, many aspects of the reproductive biology of halophytes remain to be elucidated. An important step in this process is the recent genome sequencing of *Z. marina* (Olsen et al., 2016) and *Chenopodium quinoa* (Jarvis et al., 2017; Zou et al., 2017). In the near future, it will be necessary to obtain more genome sequences so as to study salt-tolerance mechanisms in a broader range of halophytes – such as *L. bicolor*, which has salt glands; and *S. salsa*, which shows leaf and stem succulence – to detect the key genes and networks underlying these traits.

Halophytes provide good material for studying salt-tolerance mechanisms, especially those involved in seed formation and germination under saline conditions. Subsequent salt-tolerance studies may thus be focused on halophytes instead of non-halophytes. Moreover, mutant libraries for halophytes should be constructed to verify the functions of salt-tolerance genes. The use of ethyl methanesulfonate (EMS) or gamma ray mutagenesis and/or CRISPR-cas9 in halophytes may represent feasible approaches to obtaining mutants with differences in salt tolerance (Yuan et al., 2013, 2015). We hope to obtain salt-sensitive halophyte strains by applying the above mutagenesis techniques followed by transcriptome or expression profiling. Such work would allow us to accurately identify a series of salt-related genes by comparing the mutants’ profiles with those of the wild type. Therefore, more efficient transformation systems for different types of halophytes urgently need to be established. The detailed mechanisms underlying the good reproductive growth and early germination of halophytes can then be further investigated at the molecular level.

## Author Contributions

BW designed the manuscript. FY and JG wrote the manuscript. BW and SS revised the manuscript.

## Conflict of Interest Statement

The authors declare that the research was conducted in the absence of any commercial or financial relationships that could be construed as a potential conflict of interest.

## References

[B1] Abdullah Z. Khan M. A. Flowers T. J. (2010). Causes of sterility in seed set of rice under salinity stress. *J. Agron. Crop Sci.* 187 25–32. 10.1046/j.1439-037X.2001.00500.x

[B2] Ayarpadikannan S. Chung E. Cho C. W. So H. A. Kim S. O. Jeon J. M. (2012). Exploration for the salt stress tolerance genes from a salt-treated halophyte, Suaeda asparagoides. *Plant Cell Rep.* 31 35–48. 10.1007/s00299-011-1137-4 21874516

[B3] Ben Amor N. Jimenez A. Megdiche W. Lundqvist M. (2010). Response of antioxidant systems to NaCl stress in the halophyte *Cakile maritima*. *Physiol. Plant.* 126 446–457. 10.1111/j.1399-3054.2006.00620.x

[B4] Blázquez M. A. Ferrándiz C. Madueño F. Parcy F. (2006). How floral meristems are built. *Plant Mol. Biol.* 60 855–870. 10.1007/s11103-006-0013-z 16724257

[B5] Cao D. Baskin C. C. Baskin J. M. Yang F. Huang Z. (2012). Comparison of germination and seed bank dynamics of dimorphic seeds of the cold desert halophyte *Suaeda corniculata* subsp. *mongolica. Ann. Bot.* 110 1545–1548. 10.1093/aob/mcs205 22975287 PMC3503492

[B6] Cao Y. R. Chen S. Y. Zhang J. S. (2008). Ethylene signaling regulates salt stress response. *Plant Signal. Behav.* 3 761–763. 10.4161/psb.3.10.5934 19513226 PMC2634369

[B7] Chang W. Liu X. Zhu J. Fan W. Zhang Z. (2016). An aquaporin gene from halophyte *Sesuvium portulacastrum*, SpAQP1, increases salt tolerance in transgenic tobacco. *Plant Cell Rep.* 35 385–395. 10.1007/s00299-015-1891-9 26581952

[B8] Chen M. Song J. Wang B. S. (2010). NaCl increases the activity of the plasma membrane H^+^-ATPase in C3 halophyte *Suaeda salsa* callus. *Acta Physiol. Plant.* 32 27–36. 10.1007/s11738-009-0371-7

[B9] Chen T. S. Yuan F. Song J. Wang B. S. (2016). Nitric oxide participates in waterlogging tolerance through enhanced adventitious root formation in the euhalophyte *Suaeda salsa*. *Funct. Plant Biol.* 43 244–253. 10.1071/FP15120 32480457

[B10] Chen Y. Li L. Zong J. Chen J. Guo H. Guo A. (2015). Heterologous expression of the halophyte *Zoysia matrella* H^+^-pyrophosphatase gene improved salt tolerance in *Arabidopsis thaliana*. *Plant Physiol. Biochem.* 91 49–55. 10.1016/j.plaphy.2015.04.004 25874657

[B11] Chen Z. Pottosin I. I. Cuin T. A. Fuglsang A. T. Tester M. Jha D. (2007). Root plasma membrane transporters controlling K^+^/Na^+^ homeostasis in salt-stressed barley. *Plant Physiol.* 145 1714–1725. 10.1104/pp.107.110262 17965172 PMC2151677

[B12] Chrominski A. Bhat R. B. Weber D. J. Smith B. N. (1988). Osmotic stress-dependent conversion of 1-aminocyclopropane-1-carboxylic acid (ACC) to ethylene in the halophyte, *Allenrolfea occidentalis*. *Environ. Exp. Bot.* 28 171–174. 10.1016/0098-8472(88)90026-3

[B13] Chrominski A. Halls S. Weber D. J. Smith B. N. (1989). Proline affects ACC to ethylene conversion under salt and water stresses in the halophyte, *Allenrolfea occidentalis*. *Environ. Exp. Bot.* 29 359–363. 10.1016/0098-8472(89)90010-5

[B14] Chrominski A. Weber D. J. Smith B. N. Khan A. M. (1986). NaCl-salinity-dependent conversion of ACC to ethylene in the halophyte, *Allenrolfea occidentalis*. *Naturwissenschaften* 73 274–275. 10.1007/BF00367785

[B15] Cockram J. Jones H. Leigh F. J. O’sullivan D. Powell W. Laurie D. A. (2007). Control of flowering time in temperate cereals: genes, domestication, and sustainable productivity. *J. Exp. Bot.* 58 1231–1244. 10.1093/jxb/erm042 17420173

[B16] Dassanayake M. Larkin J. C. (2017). Making plants break a sweat: the structure, function, and evolution of plant salt glands. *Front. Plant Sci.* 8:406. 10.3389/fpls.2017.00406 28400779 PMC5368257

[B17] Debez A. Hamed K. B. Grignon C. Abdelly C. (2004). Salinity effects on germination, growth, and seed production of the halophyte *Cakile maritima*. *Plant Soil* 262 179–189. 10.1023/B:PLSO.0000037034.47247.67

[B18] Demidchik V. Tester M. (2002). Sodium fluxes through nonselective cation channels in the plasma membrane of protoplasts from Arabidopsis roots. *Plant Physiol.* 128 379–387. 10.1104/pp.010524 11842142 PMC148901

[B19] Ding F. Chen M. Sui N. Wang B. S. (2010a). Ca^2+^ significantly enhanced development and salt-secretion rate of salt glands of *Limonium bicolor* under NaCl treatment. *S. Afr. J. Bot.* 76 95–101. 10.1016/j.sajb.2009.09.001

[B20] Ding F. Yang J. C. Yuan F. Wang B. S. (2010b). Progress in mechanism of salt excretion in recretohalopytes. *Front. Biol.* 5 164–170. 10.1007/s11515-010-0032-7

[B21] Feng Z. T. Deng Y. Q. Zhang S. C. Liang X. Yuan F. Hao J. L. (2015). K^+^ accumulation in the cytoplasm and nucleus of the salt gland cells of *Limonium bicolor* accompanies increased rates of salt secretion under NaCl treatment using NanoSIMS. *Plant Sci.* 238 286–296. 10.1016/j.plantsci.2015.06.021 26259195

[B22] Feng Z. T. Sun Q. J. Deng Y. Q. Sun S. F. Zhang J. G. Wang B. S. (2014). Study on pathway and characteristics of ion secretion of salt glands of *Limonium bicolor*. *Acta Physiol. Plant.* 36 2729–2741. 10.1007/s11738-014-1644-3

[B23] Flowers T. J. Colmer T. D. (2008). Salinity tolerance in halophytes. *New Phytol.* 179 945–963. 10.1111/j.1469-8137.2008.02531.x 18565144

[B24] Flowers T. J. Galal H. K. Bromham L. (2010). Evolution of halophytes: multiple origins of salt tolerance in land plants. *Funct. Plant Biol.* 37 604–612. 10.1071/FP09269 21472467

[B25] Flowers T. J. Munns R. Colmer T. D. (2015). Sodium chloride toxicity and the cellular basis of salt tolerance in halophytes. *Ann. Bot.* 115 419–431. 10.1093/aob/mcu217 25466549 PMC4332607

[B26] Galvan-Ampudia C. Christa T. (2011). Salt stress signals shape the plant root. *Curr. Opin. Plant Biol.* 14 296–302. 10.1016/j.pbi.2011.03.019 21511515

[B27] Gasparri R. Casavecchia S. Galié M. Pesaresi S. Soriano P. Estrelles E. (2016). Germination pattern of Salicornia patula as an adaptation to environmental conditions of the specific populations. *Plant Sociol.* 53 91–104.

[B28] Glenn E. P. Jed Brown J. O’leary J. W. (1998). Irrigating crops with seawater. *Sci. Am.* 279 76–81. 10.1038/scientificamerican0898-76 24222734

[B29] Gul B. Ansari R. Flowers T. J. Khan M. A. (2013). Germination strategies of halophyte seeds under salinity. *Environ. Exp. Bot.* 92 4–18. 10.1016/j.envexpbot.2012.11.006

[B30] Gunning D. (2016). *Cultivating Salicornia europaea (Marsh Samphire).* Dublin: Irish Sea Fisheries Board.

[B31] Guo J. Li Y. Han G. Song J. Wang B. S. (2018). NaCl markedly improved the reproductive capacity of the euhalophyte *Suaeda salsa*. *Funct. Plant Biol.* 44 350–361. 10.1071/FP17181 32290958

[B32] Guo J. Suo S. Wang B. S. (2015). Sodium chloride improves seed vigour of the euhalophyte *Suaeda salsa*. *Seed Sci. Res.* 25 335–344. 10.1017/S0960258515000239

[B33] Guo S. Yin H. Zhang X. Zhao F. Li P. Chen S. (2006). Molecular cloning and characterization of a vacuolar H^+^-pyrophosphatase gene, SsVP, from the halophyte *Suaeda salsa* and its overexpression increases salt and drought tolerance of *Arabidopsis*. *Plant Mol. Biol.* 60 41–50. 10.1007/s11103-005-2417-6 16463098

[B34] Guo Y. H. Jia W. J. Song J. Wang D. Chen M. Wang B. S. (2012a). *Thellungilla halophila* is more adaptive to salinity than *Arabidopsis thaliana* at stages of seed germination and seedling establishment. *Acta Physiol. Plant.* 34 1287–1294. 10.1007/s11738-012-0925-y

[B35] Guo Y. H. Wang D. Jia W. J. Song J. Yang J. C. Wang B. S. (2012b). Effects of seed vernalisation and photoperiod on flowering induction in the halophyte *Thellungiella halophila*. *Aust. J. Bot.* 60 743–748. 10.1071/BT12180

[B36] Himabindu Y. Chakradhar T. Reddy M. C. Kanygin A. Redding K. E. Chandrasekhar T. (2016). Salt-tolerant genes from halophytes are potential key players of salt tolerance in glycophytes. *Environ. Exp. Bot.* 124 39–63. 10.1016/j.envexpbot.2015.11.010

[B37] Ishimaru K. (1999). Transformation of a CAM plant, the facultative halophyte *Mesembryanthemum crystallinum* by *Agrobacterium tumefaciens*. *Plant Cell Tissue Organ Cult.* 57 61–63. 10.1023/A:1006225212298

[B38] Jarvis D. E. Ho Y. S. Lightfoot D. J. Schmöckel S. M. Li B. Tja B. (2017). The genome of *Chenopodium quinoa*. *Nature* 542 307–312. 10.1038/nature21370 28178233

[B39] Jha B. Lal S. Tiwari V. Yadav S. K. Agarwal P. K. (2012). The *SbASR* -1 gene cloned from an extreme halophyte *Salicornia brachiata* enhances salt tolerance in transgenic tobacco. *Mar. Biotechnol.* 14 782–792. 10.1007/s10126-012-9442-7 22639284

[B40] Jia G. Liu X. Owen H. A. Zhao D. (2008). Signaling of cell fate determination by the TPD1 small protein and EMS1 receptor kinase. *Proc. Natl. Acad. Sci. U.S.A.* 105 2220–2225. 10.1073/pnas.0708795105 18250314 PMC2538901

[B41] Jiang A. M. Lu G. Yi T. Ma H. X. Zhang J. M. Song Z. J. (2013). The effect of genome duplication on seed germination and seedling growth of rice under salt stress. *Aust. J. Crop Sci.* 7 1814–1821.

[B42] Jiang Y. Zhang L. Gu D. Zhang Q. Xue F. Li Y. (2014). Effect of salt stress on seed germination of *Impatiens balsamina* L. *J. Northeast For. Univ.* 42 37–41.

[B43] Jung J. Park C. (2011). Auxin modulation of salt stress signaling in Arabidopsis seed germination. *Plant Signal. Behav.* 6 1198–1200. 10.4161/psb.6.8.15792 21757997 PMC3260721

[B44] Keblawy A. E. Neyadi S. S. A. Rao M. V. Almarzouqi A. H. (2011). Interactive effects of salinity, light and temperature on seed germination of sand dunes glycophyte *Cyprus conglomeratus* growing in the United Arab Emirates deserts. *Seed Sci. Technol.* 39 364–376. 10.15258/sst.2011.39.2.09

[B45] Keiffer C. H. Ungar I. A. (1997). The effect of extended exposure to hypersaline conditions on the germination of five inland halophyte species. *Am. J. Bot.* 84 104–111. 10.2307/2445887

[B46] Khan H. A. Siddique K. H. Munir R. Colmer T. D. (2015). Salt sensitivity in chickpea: growth, photosynthesis, seed yield components and tissue ion regulation in contrasting genotypes. *J. Plant Physiol.* 182 1–12. 10.1016/j.jplph.2015.05.002 26037693

[B47] Khan M. A. Ansari R. Gul B. Li W. (2009). Dormancy and germination responses of halophyte seeds to the application of ethylene. *C. R. Biol.* 332 806–815. 10.1016/j.crvi.2009.05.002 19748455

[B48] Khan M. A. Ungar I. A. (1996). Influence of salinity and temperature on the germination of *Haloxylon recurvum* Bunge ex. Boiss. *Ann. Bot.* 78 547–551. 10.1006/anbo.1996.0159

[B49] Khan M. A. Ungar I. A. (1997). Effects of thermoperiod on recovery of seed germination of halophytes from saline conditions. *Am. J. Bot.* 84 279–283. 10.2307/2446089 21712207

[B50] Khan M. A. Ungar I. A. Showalter A. M. (2000). The effect of salinity on the growth, water status, and ion content of a leaf succulent perennial halophyte, *Suaeda fruticosa* (L.) Forssk. *J. Arid Environ.* 45 73–84. 10.1006/jare.1999.0617

[B51] Khatun S. Flowers T. J. (1995). Effects of salinity on seed set in rice. *Plant Cell Environ.* 18 61–67. 10.1111/j.1365-3040.1995.tb00544.x

[B52] Kim S. G. Park C. M. (2007). Membrane-mediated salt stress signaling in flowering time control. *Plant Signal. Behav.* 2 517–518. 10.4161/psb.2.6.4645 19704545 PMC2634355

[B53] Kirch H. H. Veraestrella R. Golldack D. Quigley F. Michalowski C. B. Barkla B. J. (2000). Expression of water channel proteins in *Mesembryanthemum crystallinum*. *Plant Physiol.* 123 111–124. 10.1104/pp.123.1.111 10806230 PMC58987

[B54] Komeda Y. (2004). Genetic regulation of time to flower in *Arabidopsis thaliana*. *Annu. Rev. Plant Biol.* 55 521–535. 10.1146/annurev.arplant.55.031903.141644 15377230

[B55] Krishnamurthy P. Jyothi-Prakash P. A. Qin L. Jie H. E. Lin Q. Loh C. S. (2014). Role of root hydrophobic barriers in salt exclusion of a mangrove plant *Avicennia officinalis*. *Plant Cell Environ.* 37 1656–1671. 10.1111/pce.12272 24417377

[B56] Ksouri R. Megdiche W. Debez A. Falleh H. Grignon C. Abdelly C. (2007). Salinity effects on polyphenol content and antioxidant activities in leaves of the halophyte *Cakile maritima*. *Plant Physiol. Biochem.* 45 244–249. 10.1016/j.plaphy.2007.02.001 17408958

[B57] Kwiatkowska D. (2008). Flowering and apical meristem growth dynamics. *J. Exp. Bot.* 59 187–201. 10.1093/jxb/erm290 18256052

[B58] Ledesma N. Sugiyama N. (2005). Pollen quality and performance in strawberry plants exposed to high-temperature stress. *J. Am. Soc. Hortic. Sci.* 130 341–347.

[B59] Lee I. Aukerman M. J. Gore S. L. Lohman K. N. Michaels S. D. Weaver L. M. (1994). Isolation of LUMINIDEPENDENS: a gene involved in the control of flowering time in Arabidopsis. *Plant Cell* 6 75–83. 10.1105/tpc.6.1.75 7907507 PMC160417

[B60] Lee J. S. (2011). *AGAMOUS-LIKE 6* is a floral promoter that negatively regulates the *FLC/MAF* clade genes and positively regulates *FT* in Arabidopsis. *Plant J.* 65 62–76. 10.1111/j.1365-313X.2010.04402.x 21175890

[B61] Leng B. Y. Yuan F. Dong X. X. Wang J. Wang B. S. (2018). Distribution pattern and salt excretion rate of salt glands in two recretohalophyte species of *Limonium* (Plumbaginaceae). *S. Afr. J. Bot.* 115 74–80. 10.1016/j.sajb.2018.01.002

[B62] Li K. Pang C. H. Ding F. Sui N. Feng Z. T. Wang B. S. (2012). Overexpression of *Suaeda salsa* stroma ascorbate peroxidase in *Arabidopsis* chloroplasts enhances salt tolerance of plants. *S. Afr. J. Bot.* 78 235–245. 10.1016/j.sajb.2011.09.006

[B63] Li X. Liu Y. Chen M. Song Y. P. Song J. Wang B. S. (2012). Relationships between ion and chlorophyll accumulation in seeds adaptation to saline environments in *Suaeda salsa* populations. *G. Bot. Ital.* 146 142–149. 10.1080/11263504.2012.727880

[B64] Li W. An P. Liu X. (2008). Effect of storage, stratification, temperature and gibberellins on germination of dimorphic seeds of *Suaeda salsa* under saline conditions. *Seed Sci. Technol.* 36 122–132. 10.15258/sst.2008.36.1.13

[B65] Li W. Tran L. S. P. (2017). Effects of ethylene on seed germination of halophyte plants under salt stress. *Methods Mol. Biol.* 1573 253–259. 10.1007/978-1-4939-6854-1_18 28293852

[B66] Li W. Wang D. Jin T. Chang Q. Yin D. Xu S. (2011). The vacuolar Na^+^/H^+^ antiporter gene *SsNHX1* from the halophyte *Salsola soda* confers salt tolerance in transgenic alfalfa (*Medicago sativa* L.). *Plant Mol. Biol. Rep.* 29 278–290. 10.1007/s11105-010-0224-y

[B67] Li X. Zhang X. Song J. Fan H. Feng G. Wang B. (2011). Accumulation of ions during seed development under controlled saline conditions of two *Suaeda salsa* populations is related to their adaptation to saline environments. *Plant Soil* 341 99–107. 10.1007/s11104-010-0625-6

[B68] Lin Y. Lei Y. Chen D. Zu Y. Tang Z. (2013). A role for Ethylene-Insensitive3 in the regulation of hydrogen peroxide production during seed germination under high salinity in *Arabidopsis*. *Acta Physiol. Plant.* 35 1701–1706. 10.1007/s11738-012-1176-7

[B69] Liu L. Zeng Y. Pan X. Zhang F. (2012). Isolation, molecular characterization, and functional analysis of the vacuolar Na^+^/H^+^ antiporter genes from the halophyte *Karelinia caspica*. *Mol. Biol. Rep.* 39 7193–7202. 10.1007/s11033-012-1551-x 22311041

[B70] Liu Q. Liu R. Ma Y. Song J. (2018). Physiological and molecular evidence for Na^+^ and Cl- exclusion in the roots of two *Suaeda salsa* populations. *Aquat. Bot.* 146 1–7. 10.1016/j.aquabot.2018.01.001

[B71] Liu R. Wang L. Tanveer M. Song J. (2018). Seed heteromorphism: an important adaptation of halophytes for habitat heterogeneity. *Front. Plant Sci.* 9:1515. 10.3389/fpls.2018.01515 30386364 PMC6199896

[B72] Ma X. Qiao Z. Chen D. Yang W. Zhou R. Zhang W. (2015). CYCLIN-DEPENDENT KINASE G2 regulates salinity stress response and salt mediated flowering in *Arabidopsis thaliana*. *Plant Mol. Biol.* 88 287–299. 10.1007/s11103-015-0324-z 25948280

[B73] Macrobbie E. A. C. (2000). ABA activates multiple Ca^2+^ fluxes in stomatal guard cells, triggering vacuolar K^+^(Rb^+^) release. *Proc. Natl. Acad. Sci. U.S.A.* 97 12361–12368. 10.1073/pnas.220417197 11027317 PMC17347

[B74] Magome H. Yamaguchi S. Hanada A. Kamiya Y. Oda K. (2008). The DDF1 transcriptional activator upregulates expression of a gibberellin-deactivating gene, *GA2ox7*, under high-salinity stress in Arabidopsis. *Plant J.* 56 613–626. 10.1111/j.1365-313X.2008.03627.x 18643985

[B75] Malcolm C. V. Lindley V. A. O’leary J. W. Runciman H. V. Barrettlennard E. G. (2003). Halophyte and glycophyte salt tolerance at germination and the establishment of halophyte shrubs in saline environments. *Plant Soil* 253 171–185. 10.1023/A:1024578002235

[B76] Manousaki E. Kalogerakis N. (2011). Halophytes present new opportunities in phytoremediation of heavy metals and saline soils. *Ind. Eng. Chem. Res.* 50 656–660. 10.1021/ie100270x

[B77] Mario B. A. Zhu J. K. (2009). SIK1/SOS2 networks: decoding sodium signals via calcium-responsive protein kinase pathways. *Pflugers Arch.* 458 613–619. 10.1007/s00424-009-0646-2 19247687 PMC2691526

[B78] Meot-Duros L. Magné C. (2008). Effect of salinity and chemical factors on seed germination in the halophyte *Crithmum maritimum* L. *Plant Soil* 313 83–87. 10.1007/s11104-008-9681-6

[B79] Monteiro S. Piçarrapereira M. A. Teixeira A. R. Loureiro V. B. Ferreira R. B. (2003). Environmental conditions during vegetative growth determine the major proteins that accumulate in mature grapes. *J. Agric. Food Chem.* 51 4046–4053. 10.1021/jf020456v 12822945

[B80] Mori I. C. Schroeder J. I. (2004). Reactive oxygen species activation of plant Ca^2+^ channels. A signaling mechanism in polar growth, hormone transduction, stress signaling, and hypothetically mechanotransduction. *Plant Physiol.* 135 702–708. 10.1104/pp.104.042069 15208417 PMC514107

[B81] Nakaune M. Hanada A. Yin Y. G. Matsukura C. Yamaguchi S. Ezura H. (2012). Molecular and physiological dissection of enhanced seed germination using short-term low-concentration salt seed priming in tomato. *Plant Physiol. Biochem.* 52 28–37. 10.1016/j.plaphy.2011.11.005 22305065

[B82] Nandwal A. S. Kukreja S. Kumar N. Sharma P. K. Jain M. Mann A. (2007). Plant water status, ethylene evolution, N2-fixing efficiency, antioxidant activity and lipid peroxidation in *Cicer arietinum* L. nodules as affected by short-term salinization and desalinization. *J. Plant Physiol.* 164 1161–1169. 10.1016/j.jplph.2006.05.017 16987567

[B83] Neves G. Y. S. Zonetti P. D. C. Ferrarese M. D. L. L. Braccini A. D. L. E. Ferraresefilho O. (2005). Seed germination and seedlings growth of soybean (*Glycine max* (L.) Merr.) under salt stress. *Biosci. J.* 21 77–83.

[B84] Nico D. S. Danny G. (2014). The impact of environmental stress on male reproductive development in plants: biological processes and molecular mechanisms. *Plant Cell Environ.* 37 1–18. 10.1111/pce.12142 23731015 PMC4280902

[B85] Niranjan B. Ramanarao M. V. Kanniah R. Prasanta S. Jaroslav J. David G. (2012). Enhanced salt stress tolerance of rice plants expressing a vacuolar H^+^-ATPase subunit c1 (*SaVHAc1*) gene from the halophyte grass *Spartina alterniflora* Löisel. *Plant Biotechnol. J.* 10 453–464. 10.1111/j.1467-7652.2012.00678.x 22284568

[B86] Oh D. Barkla B. Vera-Estrella R. Pantoja O. Lee S. Bohnert H. (2015). Cell type-specific responses to salinity-the epidermal bladder cell transcriptome of *Mesembryanthemum crystallinum*. *New Phytol.* 207 627–644. 10.1111/nph.13414 25944243

[B87] Olsen J. L. Rouzé P. Verhelst B. Lin Y. C. Bayer T. Collen J. (2016). The genome of the seagrass *Zostera marina* reveals angiosperm adaptation to the sea. *Nature* 530 331–335. 10.1038/nature16548 26814964

[B88] Orlovsky N. S. Japakova U. N. Zhang H. F. Volis S. (2016). Effect of salinity on seed germination, growth and ion content in dimorphic seeds of *Salicornia europaea* L. (*Chenopodiaceae*). *Plant Divers.* 38 183–189. 10.1016/j.pld.2016.06.005 30159463 PMC6112202

[B89] Ozfidankonakci C. Uzilday B. Ozgur R. Yildiztugay E. Sekmen A. H. Turkan I. (2016). Halophytes as a source of salt tolerance genes and mechanisms: a case study for the Salt Lake area, Turkey. *Funct. Plant Biol.* 43 575–589. 10.1071/FP15288 32480488

[B90] Papastylianou P. Bakogianni N. N. Travlos I. Roussis I. (2017). Sensitivity of seed germination to salt stress in black cumin (*Nigella sativa* L.). *Not. Bot. Horti Agrobot. Cluj Napoca* 46 202–205. 10.15835/nbha46110861

[B91] Parish R. W. Li S. F. (2010). Death of a tapetum: a programme of developmental altruism. *Plant Sci.* 178 73–89. 10.1016/j.plantsci.2009.11.001

[B92] Piskurewicz U. Jikumaru Y. Kinoshita N. Nambara E. Kamiya Y. Lopezmolina L. (2008). The gibberellic acid signaling repressor RGL2 inhibits *Arabidopsis* seed germination by stimulating abscisic acid synthesis and ABI5 activity. *Plant Cell* 20 2729–2745. 10.1105/tpc.108.061515 18941053 PMC2590721

[B93] Plackett A. R. G. Thomas S. G. Wilson Z. A. Hedden P. (2011). Gibberellin control of stamen development: a fertile field. *Trends Plant Sci.* 16 568–578. 10.1016/j.tplants.2011.06.007 21824801

[B94] Pujol J. A. Calvo J. F. Ramírez-Díaz L. (2000). Recovery of germination from different osmotic conditions by four halophytes from southeastern spain. *Ann. Bot.* 85 279–286. 10.1006/anbo.1999.1028

[B95] Qu X. X. Huang Z. Y. Baskin J. M. Baskin C. C. (2007). Effect of temperature, light and salinity on seed germination and radicle growth of the geographically widespread halophyte shrub *Halocnemum strobilaceum*. *Ann. Bot.* 101 293–299. 10.1093/aob/mcm047 17428834 PMC2711011

[B96] Rengel Z. (1992a). Review: the role of calcium in salt toxicity. *Plant Cell Environ.* 15 625–632. 10.1111/j.1365-3040.1992.tb01004.x

[B97] Rengel Z. (1992b). The role of calcium in salt toxicity. *Plant Cell Environ.* 15 625–632. 10.1111/j.1365-3040.1992.tb01004.x

[B98] Richards R. A. (1992). Increasing salinity tolerance of grain crops: is it worthwhile? *Plant Soil* 146 89–98. 10.1007/BF00012000

[B99] Rolletschek H. Weber H. Borisjuk L. (2003). Energy status and its control on embryogenesis of legumes. Embryo photosynthesis contributes to oxygen supply and is coupled to biosynthetic fluxes. *Plant Physiol.* 132 1196–1206. 10.1104/pp.102.017376 12857802 PMC167060

[B100] Rozema J. Flowers T. (2008). Crops for a salinized world. *Science* 322 1478–1480. 10.1126/science.1168572 19056965

[B101] Ruan C. J. Teixeira da Silva J. A. Susan M. Qin P. Stanley L. (2010). Halophyte improvement for a salinized world. *Crit. Rev. Plant Sci.* 29 329–359. 10.1080/07352689.2010.524517

[B102] Ruiz-García L. Madueño F. Wilkinson M. Haughn G. Salinas J. Martínez-Zapater J. M. (1997). Different roles of flowering-time genes in the activation of floral initiation genes in Arabidopsis. *Plant Cell* 9 1921–1934. 10.1105/tpc.9.11.1921 9401118 PMC157047

[B103] Ryu J. Y. Lee H. J. Seo P. J. Jung J. H. Ahn J. H. Park C. M. (2014). The *Arabidopsis* floral repressor BFT delays flowering by competing with FT for FD binding under high salinity. *Mol. Plant* 7 377–387. 10.1093/mp/sst114 23935007

[B104] Sakuraba Y. Bülbül S. Piao W. Choi G. Paek N. C. (2017). Arabidopsis *EARLY FLOWERING3* increases salt tolerance by suppressing salt stress response pathways. *Plant J.* 92 1106–1120. 10.1111/tpj.13747 29032592

[B105] Santos J. Al-Azzawi M. Aronson J. Flowers T. J. (2015). eHALOPH a database of salt-tolerant plants: helping put halophytes to work. *Plant Cell Physiol.* 57:e10. 10.1093/pcp/pcv155 26519912

[B106] Shabala L. Mackay A. Tian Y. Jacobsen S. E. Zhou D. Shabala S. (2012). Oxidative stress protection and stomatal patterning as components of salinity tolerance mechanism in quinoa (*Chenopodium quinoa*). *Physiol. Plant.* 146 26–38. 10.1111/j.1399-3054.2012.01599.x 22324972

[B107] Shabala S. (2013). Learning from halophytes: physiological basis and strategies to improve abiotic stress tolerance in crops. *Ann. Bot.* 112 1209–1221. 10.1093/aob/mct205 24085482 PMC3806534

[B108] Shabala S. Bose J. Hedrich R. (2014). Salt bladders: do they matter? *Trends Plant Sci.* 19 687–691. 10.1016/j.tplants.2014.09.001 25361704

[B109] Shabala S. Cuin T. A. Pottosin I. (2007). Polyamines prevent NaCl-induced K^+^ efflux from pea mesophyll by blocking non-selective cation channels. *FEBS Lett.* 581 1993–1999. 10.1016/j.febslet.2007.04.032 17467698

[B110] Shao Q. Han N. Ding T. Zhou F. Wang B. S. (2014). SsHKT1;1 is a potassium transporter of a C3 halophyte *Suaeda salsa* involving in salt tolerance. *Funct. Plant Biol.* 41 790–802. 10.1071/FP13265 32481033

[B111] Shu K. Qi Y. Chen F. Meng Y. Luo X. Shuai H. (2017). Salt stress represses soybean seed germination by negatively regulating GA biosynthesis while positively mediating ABA biosynthesis. *Front. Plant Sci.* 8:1372. 10.3389/fpls.2017.01372 28848576 PMC5554363

[B112] Singh N. Mishra A. Jha B. (2014). Over-expression of the peroxisomal ascorbate peroxidase (SbpAPX) gene cloned from halophyte *Salicornia brachiata* confers salt and drought stress tolerance in transgenic tobacco. *Mar. Biotechnol.* 16 321–332. 10.1007/s10126-013-9548-6 24197564

[B113] Sohrabi Y. Heidari G. Esmailpoor B. (2008). Effect of salinity on growth and yield of Desi and Kabuli chickpea cultivars. *Pak. J. Biol. Sci.* 11 664–667. 10.3923/pjbs.2008.664.667 18817146

[B114] Sohrabikertabad S. Ghanbari A. Mohassel M. Mohamad H. R. Mahalati M. N. Gherekhloo J. (2013). Effect of desiccation and salinity stress on seed germination and initial plant growth of *Cucumis melo*. *Planta Daninha* 31 833–841. 10.1590/S0100-83582013000400009

[B115] Sokolovski S. Hills A. Gay R. A. Blatt M. R. (2008). Functional interaction of the SNARE protein NtSyp121 in Ca^2+^ channel gating, Ca^2+^ transients and ABA signalling of stomatal guard cells. *Mol. Plant* 1 347–358. 10.1093/mp/ssm029 19825544

[B116] Song J. Fan H. Zhao Y. Jia Y. Du X. Wang B. (2008). Effect of salinity on germination, seedling emergence, seedling growth and ion accumulation of a euhalophyte *Suaeda salsa* in an intertidal zone and on saline inland. *Aquat. Bot.* 88 331–337. 10.1016/j.aquabot.2007.11.004

[B117] Song J. Feng G. Tian C. Zhang F. (2005). Strategies for adaptation of *Suaeda physophora*, *Haloxylon ammodendron* and *Haloxylon persicum* to a saline environment during seed-germination stage. *Ann. Bot.* 96 399–405. 10.1093/aob/mci196 16002418 PMC4246778

[B118] Song J. Shi G. Gao B. Fan H. Wang B. (2011). Waterlogging and salinity effects on two *Suaeda salsa* populations. *Physiol. Plant.* 141 343–351. 10.1111/j.1399-3054.2011.01445.x 21214881

[B119] Song J. Shi W. Liu R. Xu Y. Sui N. Zhou J. (2017). The role of the seed coat in adaptation of dimorphic seeds of the euhalophyte *Suaeda salsa* to salinity. *Plant Species Biol.* 32 107–114. 10.1111/1442-1984.12132

[B120] Song J. Wang B. (2015). Using euhalophytes to understand salt tolerance and to develop saline agriculture: *Suaeda salsa* as a promising model. *Ann. Bot.* 115 541–553. 10.1093/aob/mcu194 25288631 PMC4332605

[B121] Song J. Zhou J. Zhao W. Xu H. Wang F. Xu Y. (2016). Effects of salinity and nitrate on production and germination of dimorphic seeds plant applied both through the mother in exogenously during germination *Suaeda salsa*. *Plant Species Biol.* 31 19–28. 10.1111/1442-1984.12071

[B122] Sui N. Li M. Li K. Song J. Wang B.-S. (2010). Increase in unsaturated fatty acids in membrane lipids of *Suaeda salsa* L. enhances protection of photosystem II under high salinity. *Photosynthetica* 48 623–629. 10.1007/s11099-010-0080-x

[B123] Sulpice R. Tsukaya H. Nonaka H. Mustardy L. Chen T. H. Murata N. (2003). Enhanced formation of flowers in salt-stressed *Arabidopsis* after genetic engineering of the synthesis of glycine betaine. *Plant J.* 36 165–176. 10.1046/j.1365-313X.2003.01873.x 14535882

[B124] Sun Y. L. Hong S. K. (2012). *Agrobacterium tumefaciens* -mediated transformation of the halophyte *Leymus chinensis* (Trin.). *Plant Mol. Biol. Rep.* 30 1253–1263. 10.1007/s11105-012-0434-6

[B125] Sun Z. B. Qi X. Y. Wang Z. L. Li P. H. Wu C. X. Zhang H. (2013). Overexpression of TsGOLS2, a galactinol synthase, in *Arabidopsis thaliana* enhances tolerance to high salinity and osmotic stresses. *Plant Physiol. Biochem.* 69 82–89. 10.1016/j.plaphy.2013.04.009 23728391

[B126] Takeno K. Yamaguchi H. (1991). Diversity in seed germination behavior in relation to heterocarpy in *Salsola komarovii* Iljin. *Bot. Mag.* 104 207–215. 10.1007/BF02489453

[B127] Tan W. K. Lin Q. Lim T. M. Kumar P. Loh C. S. (2013). Dynamic secretion changes in the salt glands of the mangrove tree species *Avicennia officinalis* in response to a changing saline environment. *Plant Cell Environ.* 36 1410–1422. 10.1111/pce.12068 23336288

[B128] Tasaki H. (2008). Light effect on seed chlorophyll content and germination performance of tomato and muskmelon seeds. *Class. Antiq.* 19 264–303.

[B129] Tobe K. Li X. Omasa K. (2000). Seed germination and radicle growth of a halophyte, *Kalidium caspicum* (Chenopodiaceae). *Ann. Bot.* 85 391–396. 10.1006/anbo.1999.1077

[B130] Tobe K. Li X. Omasa K. (2002). Effects of sodium, magnesium and calcium salts on seed germination and radicle survival of a halophyte, *Kalidium caspicum* (Chenopodiaceae). *Aust. J. Bot.* 50 163–169. 10.1071/BT01065

[B131] Tobe K. Li X. Omasa K. (2004). Effects of five different salts on seed germination and seedling growth of *Haloxylon ammodendron* (Chenopodiaceae). *Seed Sci. Res.* 14 345–353. 10.1079/SSR2004188

[B132] Ungar I. A. (1978). Halophyte seed germination. *Bot. Rev.* 44 233–264. 10.1007/BF02919080

[B133] Ungar I. A. (1979). Seed dimorphism in *Salicornia europaea* L. *Bot. Gaz.* 140 102–108. 10.1086/337063

[B134] Verma C. M. Bohra S. P. Sankhla N. (1973). Lettuce seed germination: reversal of salinity induced inhibition by ethylene. *Curr. Sci.* 42 294–295.

[B135] Vicente O. Boscaiu M. Naranjo M. Estrelles E. Bellés J. M. A. Soriano P. (2004). Responses to salt stress in the halophyte *Plantago crassifolia* (Plantaginaceae). *J. Arid Environ.* 58 463–481. 10.1016/j.jaridenv.2003.12.003 27490924

[B136] Wang F. Xu Y. G. Wang S. Shi W. Liu R. Feng G. (2015). Salinity affects production and salt tolerance of dimorphic seeds of *Suaeda salsa*. *Plant Physiol. Biochem.* 95 41–48. 10.1016/j.plaphy.2015.07.005 26184090

[B137] Wang F. X. Yin C. H. Song Y. P. Li Q. Tian C. Y. Song J. (2017). Reproductive allocation and fruit-set pattern in the euhalophyte *Suaeda salsa* in controlled and field conditions. *Plant Biosyst.* 152 749–758. 10.1080/11263504.2017.1330776

[B138] Wang H. L. Wang L. Tian C. Y. Huang Z. Y. (2012). Germination dimorphism in *Suaeda acuminata*: a new combination of dormancy types for heteromorphic seeds. *S. Afr. J. Bot.* 78 270–275. 10.1016/j.sajb.2011.05.012

[B139] Wang L. Huang Z. Baskin C. C. Baskin J. M. Dong M. (2008). Germination of dimorphic seeds of the desert annual halophyte *Suaeda aralocaspica* (Chenopodiaceae), a C4 plant without kranz anatomy. *Ann. Bot.* 102 757–769. 10.1093/aob/mcn158 18772148 PMC2712381

[B140] Weber H. Borisjuk L. Wobus U. (2005). Molecular physiology of legume seed development. *Annu. Rev. Plant Biol.* 56 253–279. 10.1146/annurev.arplant.56.032604.144201 15862096

[B141] Wertis B. A. Ungar I. A. (1986). Seed demography and seedling survival in a population of *Atriplex triangularis* willd. *Am. Midl. Nat.* 116 152–162. 10.2307/2425947

[B142] Wilson R. L. Kim H. Bakshi A. Binder B. M. (2014). The ethylene receptors ETHYLENE RESPONSE1 and ETHYLENE RESPONSE2 have contrasting roles in seed germination of Arabidopsis during salt stress. *Plant Physiol.* 165 1353–1366. 10.1104/pp.114.241695 24820022 PMC4081342

[B143] Xu F. Rong X. Huang X. Cheng S. (2012). Recent advances of flowering locus T gene in higher plants. *Int. J. Mol. Sci.* 13 3773–3781. 10.3390/ijms13033773 22489182 PMC3317742

[B144] Xu Y. Liu R. Sui N. Shi W. Wang L. Tian C. (2016). Changes in endogenous hormones and seed-coat phenolics during seed storage of two *Suaeda salsa* populations. *Aust. J. Bot.* 64 325–332. 10.1071/BT16014

[B145] Xu Y. Zhao Y. Duan H. Sui N. Yuan F. Song J. (2017). Transcriptomic profiling of genes in matured dimorphic seeds of euhalophyte *Suaeda salsa*. *BMC Genomics* 18:727. 10.1186/s12864-017-4104-9 28903734 PMC5598043

[B146] Yadav N. S. Shukla P. S. Jha A. Agarwal P. K. Jha B. (2012). The SbSOS1 gene from the extreme halophyte *Salicornia brachiata* enhances Na^+^ loading in xylem and confers salt tolerance in transgenic tobacco. *BMC Plant Biol.* 12:188. 10.1186/1471-2229-12-188 23057782 PMC3548769

[B147] Yang F. Baskin J. M. Baskin C. C. (2017). Divergence in life history traits between two populations of a seed-dimorphic halophyte in response to soil salinity. *Front. Plant Sci.* 8:1028. 10.3389/fpls.2017.01028 28670319 PMC5472680

[B148] Yang M. F. Song J. Wang B. S. (2010). Organ-specific responses of vacuolar H^+^-ATPase in the shoots and roots of C3 halophyte *Suaeda salsa* to NaCl. *J. Integr. Plant Biol.* 52 308–314. 10.1111/j.1744-7909.2010.00895.x 20377691

[B149] Yao M. Zeng Y. Liu L. Huang Y. Zhao E. Zhang F. (2012). Overexpression of the halophyte *Kalidium foliatum* H^+^-pyrophosphatase gene confers salt and drought tolerance in *Arabidopsis thaliana*. *Mol. Biol. Rep.* 39 7989–7996. 10.1007/s11033-012-1645-5 22539184

[B150] Yokoishi T. Tanimoto S. (1994). Seed germination of the halophyte *Suaeda japonica* under salt stress. *J. Plant Res.* 107 385–388. 10.1007/BF02344061 30154810

[B151] Yuan F. Chen M. Leng B. Y. Wang B. (2013). An efficient autofluorescence method for screening *Limonium bicolor* mutants for abnormal salt gland density and salt secretion. *S. Afr. J. Bot.* 88 110–117. 10.1016/j.sajb.2013.06.007

[B152] Yuan F. Chen M. Yang J. C. Leng B. Y. Wang B. S. (2014). A system for the transformation and regeneration of the recretohalophyte *Limonium bicolor*. *In Vitro Cell. Dev. Biol. Plant* 50 610–617. 10.1007/s11627-014-9611-7

[B153] Yuan F. Leng B. Y. Wang B. S. (2016a). Progress in studying salt secretion from the salt glands in recretohalophytes: how do plants secrete salt? *Front. Plant Sci.* 7:977. 10.3389/fpls.2016.00977 27446195 PMC4927796

[B154] Yuan F. Lyu M. J. A. Leng B. Y. Zhu X. G. Wang B. S. (2016b). The transcriptome of NaCl-treated *Limonium bicolor* leaves reveals the genes controlling salt secretion of salt gland. *Plant Mol. Biol.* 91 241–256. 10.1007/s11103-016-0460-0 26936070

[B155] Yuan F. Liang X. Li Y. Yin S. Wang B. (2018). Methyl jasmonate improves salinity tolerance in *Limonium bicolor* by enhancing photosynthesis and abaxial salt gland density. *Funct. Plant Biol.* 46 82–92. 10.1071/FP18120 30939260

[B156] Yuan F. Lyv M. J. Leng B. Y. Zheng G. Y. Feng Z. T. Li P. H. (2015). Comparative transcriptome analysis of developmental stages of the *Limonium bicolor* leaf generates insights into salt gland differentiation. *Plant Cell Environ.* 38 1637–1657. 10.1111/pce.12514 25651944

[B157] Yuan K. Rashotte A. M. Wysocka-Diller J. W. (2011). ABA and GA signaling pathways interact and regulate seed germination and seedling development under salt stress. *Acta Physiol. Plant.* 33 261–271. 10.1007/s11738-010-0542-6

[B158] Zhang G. H. Su Q. An L. J. Wu S. (2008). Characterization and expression of a vacuolar Na^+^/H^+^ antiporter gene from the monocot halophyte *Aeluropus littoralis*. *Plant Physiol. Biochem.* 46 117–126. 10.1016/j.plaphy.2007.10.022 18061467

[B159] Zhang H. Irving L. J. Tian Y. Zhou D. (2012). Influence of salinity and temperature on seed germination rate and the hydrotime model parameters for the halophyte, *Chloris virgata*, and the glycophyte, *Digitaria sanguinalis*. *S. Afr. J. Bot.* 78 203–210. 10.1016/j.sajb.2011.08.008

[B160] Zhang S. Song J. Wang H. Feng G. (2010). Effect of salinity on photosynthesis and chloroplasts ultrastructure in cotyledons of desiccated seeds of halophytes or xerophyte growing in central Asia. *J. Plant Ecol.* 3 259–267. 10.1093/jpe/rtq005

[B161] Zhao D. Ni W. Feng B. Han T. Petrasek M. G. Ma H. (2003). Members of the *Arabidopsis-SKP1-like* gene family exhibit a variety of expression patterns and may play diverse roles in Arabidopsis. *Plant Physiol.* 133 203–217. 10.1104/pp.103.024703 12970487 PMC196598

[B162] Zhao K. F. Song J. Fan H. Zhou S. Zhao M. (2010). Growth response to ionic and osmotic stress of NaCl in salt-tolerant and salt-sensitive maize. *J. Integr. Plant Biol.* 52 468–475. 10.1111/j.1744-7909.2010.00947.x 20537042

[B163] Zhao S. Z. Sun H. Z. Chen M. Wang B. S. (2010). Light-regulated betacyanin accumulation in euhalophyte *Suaeda salsa* calli. *Plant Cell Tissue Organ Cult.* 102 99–107. 10.1007/s11240-010-9710-z

[B164] Zhou J. C. Fu T. T. Sui N. Guo J. R. Feng G. Fan J. L. (2016). The role of salinity in seed maturation of the euhalophyte *Suaeda salsa*. *Plant Biosyst.* 150 83–90. 10.1080/11263504.2014.976294

[B165] Zhu J. K. (2016). Abiotic stress signaling and responses in plants. *Cell* 167 313–324. 10.1016/j.cell.2016.08.029 27716505 PMC5104190

[B166] Zou C. Chen A. Xiao L. Muller H. M. Ache P. Haberer G. (2017). A high-quality genome assembly of quinoa provides insights into the molecular basis of salt bladder-based salinity tolerance and the exceptional nutritional value. *Cell Res.* 27 1327–1340. 10.1038/cr.2017.124 28994416 PMC5674158

